# Levels of neuroactive steroids are elevated in those who develop first-onset depression early in pregnancy

**DOI:** 10.3389/fpsyt.2025.1557560

**Published:** 2025-05-08

**Authors:** Elizabeth S. Wenzel, Jordan C. Barone, Tory A. Eisenlohr-Moul, Suzanne Alvernaz, Beatriz Peñalver Bernabé, Luca Spiro Santovito, Ibrahim M. M. Gdyana, Graziano Pinna, Pauline M. Maki

**Affiliations:** ^1^ Department of Psychiatry and Neurobehavioral Sciences, University of Virginia, Charlottesville, VA, United States; ^2^ Department of Psychology, University of Illinois Chicago, Chicago, IL, United States; ^3^ Department of Psychiatry, University of Illinois Chicago, Chicago, IL, United States; ^4^ Department of Biomedical Engineering, University of Illinois Chicago, Chicago, IL, United States; ^5^ Center for Alcohol Research in Epigenetics (CARE), University of Illinois Chicago, Chicago, IL, United States; ^6^ Department of Obstetrics and Gynecology, University of Illinois Chicago, Chicago, IL, United States

**Keywords:** neuroactive steroids, perinatal depression, allopregnanolone, antenatal depression, pregnancy

## Abstract

**Background:**

Allopregnanolone (ALLO) plays a key role in the pathogenesis of postpartum depression. However, ALLO levels have been variably associated with depression during pregnancy. It is unknown if a pre-pregnancy history of major depressive disorder (MDD), which is associated with blunted neurosteroidogenesis, and timing of perinatal depression (PND) may influence the association between neuroactive steroids (NAS) and PND.

**Objective:**

To investigate differences in ALLO and other NAS levels during the first (T1) and second (T2) trimesters based on pre-pregnancy history of MDD and current PND.

**Methods:**

Participants completed a diagnostic test of depression, blood samples and mental health history. Ninety-eight participants contributed data in T1 and 93 in T2. Levels of ALLO, pregnanolone (PA), isoallopregnanolone (ISO), epipregnanolone (EPI), and progesterone (P4) were quantified using gas chromatography-mass spectrometry. Analyses of covariance with pairwise comparisons predicted NAS levels from categorized groups of those with: 1) no history of MDD and no PND (*never depressed*), 2) a history of MDD but no PND (*pre-pregnancy depression*), 3) a history of MDD and current PND (*recurrent depression*), and 4) no history of MDD but current PND (*perinatal-emergent depression*).

**Results:**

Across groups, there were marginally significant differences in T1 ALLO (p=.05) and ISO levels (p=.05). T1 ALLO levels were higher in the perinatal-emergent versus never depressed (p=.007) and recurrent depression (p=.05) groups. ISO levels were higher in the perinatal-emergent versus recurrent depression (p=.02) and never depressed (p=.03) groups. In T2, there were significant differences in PA levels (p=.04) and marginally significant differences in ALLO (p=.07) and ISO levels (p=.09). ALLO levels were higher in the perinatal-emergent versus recurrent depression group (p=.05), and in the never depressed (p=.05) and pre-pregnancy depression groups (p=.04) compared to recurrent depression. PA levels were higher in the perinatal-emergent depression versus never depressed (p=.02) and recurrent depression (p=.01) groups, and ISO levels were higher in the perinatal-emergent depression versus never depressed (p=.03) and recurrent depression (p=.07) groups.

**Conclusions:**

These results suggest differing NAS-related mechanisms of pathogenesis across clinical phenotypes based on pre-pregnancy history of MDD and timing of PND onset. Future research should account for these factors when investigating NAS and PND.

## Introduction

The perinatal period, including pregnancy and the first year postpartum, is characterized by large fluctuations in sex hormones and their metabolites and is a time of vulnerability to mood disorders including perinatal depression (PND) (1). More than 500,000 people in the United States develop PND, with an average prevalence of 11.5% (2, 3). Neuroactive steroid (NAS) metabolites of progesterone (P4), including allopregnanolone (ALLO), are strongly implicated in the pathogenesis of PND, though the direction of association between NAS and mood symptoms during pregnancy differs across the literature (4–10). Neurosteroids are a class of cholesterol-derived endogenous steroids that are synthesized in the brain. Neurosteroids can also be synthesized in the adrenal glands and gonads, but cross the blood-brain-barrier to act collectively with brain-derived neurosteroids as *neuroactive steroids*. NAS metabolized from P4, including ALLO (3α,5α-tetrahydroprogesterone; THP) and its isomers 3α,5β-THP (pregnanolone; PA), 3β,5α -THP (isoallopregnanolone; ISO), and 3β,5β-THP (epipregnanolone; EPI) have been associated with mood changes, and with PND specifically (7, 10–14). Each of these NAS are gamma-aminobutyric acid (GABA)-receptor type A (GABA_A_R) modulators. ALLO in particular is well-studied and generally reported to exert anxiolytic and antidepressant effects as a positive allosteric modulator at the GABA_A_R (15–17). Withdrawal from P4 and ALLO after parturition has been associated with postpartum blues and depression (PPD) (9, 18). Brexanolone, an FDA-approved intravenous infusion of exogenous ALLO rapidly and effectively treats moderate/severe PPD (19). However, the association between ALLO and antenatal depression is variable across the literature, with some studies finding lower ALLO levels in depressed individuals during pregnancy (4, 5), others finding no association between ALLO and mood (6, 20), and still others finding higher ALLO levels in depressed pregnant individuals (7, 10). Factors contributing to this variability are yet unknown, particularly in early pregnancy when levels of P4 and NAS are surging.

Major depressive disorder (MDD) is a reliable risk factor for PND, but studies have not systematically considered how it affects NAS during pregnancy. MDD is widely reported to be associated with blunted neurosteroidogenesis in non-pregnant populations, and with lower levels of ALLO compared to healthy controls (11, 12, 21). In animal models, chronic stress is associated with decreased neurosteroidogenesis and HPA-axis suppression, and disorders of chronic stress such as post-traumatic stress disorder (PTSD) are associated with blunted neurosteroidogenesis in humans (22–28). In contrast, acute stress is associated with upregulation of neurosteroidogenesis and activation of the HPA-axis (fight or flight response) (25, 29). This evidence base suggests a complex interplay between mental health history, current stress and NAS and how they may influence development of PND. For example, those with a diagnosis of PND may include both those with new-onset depression during pregnancy who have never had a depressive episode before (*perinatal-emergent depressio*n) and those who are depressed pre-pregnancy and continue to be depressed during pregnancy (*recurrent depression*). Biologically, this heterogeneity may involve differences in absolute levels of NAS, *or* differences in the response to normative pregnancy changes in NAS.

Initial evidence suggests that there may be differences in NAS levels during pregnancy in those with a history of MDD. In non-pregnant women, concentrations of ALLO, PA and P4 both before and after an oral P4 challenge were lower in women with a history of MDD compared to women who had never been depressed (30). This experimental evidence suggests that NAS may be lower before and during pregnancy among women with a history of MDD. Although no study to date has examined NAS levels in pregnancy in women with versus without pre-pregnancy MDD, three studies examined NAS levels in the third trimester and postpartum in a group of women “at-risk” for PPD based on *either* a history of depression prior to pregnancy or mild depressive and anxiety symptoms during pregnancy (6–8). One found higher levels of P4 and PA in the at-risk women compared to never depressed healthy controls (7), another found higher levels of ALLO in the at-risk women compared to never depressed healthy controls regardless of whether they had PND (8), and the third found no differences in NAS across groups (6). However, the timing of depression onset in those who developed depression was not specified, and participants were assessed in late pregnancy and the postpartum, but not in early pregnancy when steroid hormones are surging. Taken together, there is evidence to suggest that there may be differences in NAS levels during pregnancy in those who enter pregnancy with a history of MDD (that may or may not persist into pregnancy), compared to those who have no history of psychiatric disorders but become depressed during pregnancy, but no study has directly investigated the relationship between a history of MDD and timing of depression onset in PND on NAS during pregnancy.

This study sought to investigate the influence of MDD history on NAS levels during the first and second trimesters of pregnancy when hormones and NAS are typically surging. Specifically, we aimed to investigate the separate and combined influences of depression before and during pregnancy across four groups of women, including those with: 1) no prior history of MDD and no depression during early pregnancy (*never depressed*); 2) a prior history of MDD but no depression during early pregnancy (*pre-pregnancy depression only*); 3) a prior history of MDD who were also depressed in early pregnancy (*recurrent depression*); and 4) no prior history of MDD who developed depression in early pregnancy (*perinatal-emergent depression*). We hypothesized that those with a history of MDD would have blunted neurosteroidogenesis evidenced by lower levels of NAS early in pregnancy, especially if they continued to be depressed during pregnancy (*recurrent depression*), compared to those who had *perinatal-emergent* depression during pregnancy, or those who were never depressed.

## Materials and methods

### Participants

Participants were a subset of subjects enrolled in an ongoing observational, longitudinal cohort study called Moms and Mental Health (MoMent). MoMent participants were recruited from the Center for Women’s Health Outpatient Clinic at the University of Illinois Chicago (UIC), an outpatient clinic that serves primarily low-income Black and Latina women. From 2014-2017, no biological samples were collected in MoMent, but starting in 2017, gut microbiome and blood samples were obtained (31). For this substudy, we included MoMent participants with complete data on a computerized adaptive test for depression (CAD-MDD) and mental health history, and who had provided blood samples in early pregnancy. Participants were enrolled between 2017 and 2024. General inclusion criteria for MoMent participants with biological sampling included: a) pregnant; b) ≤ 16 weeks gestational age at enrollment and c) 18 years of age or older. General exclusion criteria were: a) use of antibiotics, drugs including cocaine, opioids, and amphetamines (but *not* including cannabis or alcohol), or laxatives in the past 3 months, b) use of hormone treatments for infertility, c) active diagnosis of anorexia, bulimia, or had gastric bypass surgery, or d) current use of certain prescription medications, including antidepressants. This study was in accordance with ethical standards for human experimentation and was approved by the UIC Institutional Review Board (IRB #2014-0325).

### Procedures

Study visits included in the present analysis were conducted at <16 weeks gestational age (T1) and 24–28 weeks (T2). Trained research assistants identified potentially eligible participants at their initial prenatal visit. After confirming eligibility, research assistants explained the study and obtained informed consent. Blood draws took place at the T1 and T2 visits by a trained phlebotomist, often in conjunction with provider-ordered blood draws (i.e., initial prenatal blood panel for T1, 1-hour glucose challenge for T2). In the case that the T2 blood draw occurred in conjunction with a 1-hour glucose challenge, the blood draw occurred 1-hour post-glucose challenge. Participants also completed a battery of questionnaires at each visit, including measures of depression and other mood/stress measures, a mental health history, and sociodemographics. Details follow.

### Self-report mood measures

A computerized adaptive test for mental health (CAT-MH™) was used to assess current MDD. The CAT-MH™ draws from a bank of items aligned with the Diagnostic and Statistical Manual of Mental Disorders-5 and customizes itself in real-time based on participant answers. We used the version calibrated for the perinatal period (32). The CAD-MDD is a diagnostic module of the CAT-MH™ for MDD that uses ~4 items to yield a yes/no diagnosis of MDD with high sensitivity (0.95) and specificity (0.87) compared to a 1-hour clinical-based diagnosis (33). We have previously shown that the CAD-MDD detects ~5% more cases of PND in our cohort compared to the Patient Health Questionnaire-9, which is the standard screening measure for depression in the UIC Center for Women’s Health (31).

### Neuroactive steroid analysis

Serum samples were allowed to settle for at least 30 minutes prior to centrifuging for 10 minutes at 3,000 rpm and 4°C. Serum was aliquoted into.5mL cryovials and stored at −80°C until time of NAS analyses. We measured levels of P4, ALLO, PA, ISO, and EPI, examining absolute levels of each (in ng/ml). Extraction, high performance liquid chromatography (HPLC), derivatization, and gas chromatography-mass spectrometry (GC-MS) quantification analyses of NAS were performed as previously described (15, 34). Samples were extracted in ethyl acetate and lyophilized. Steroids of interest were purified and separated using HPLC. Tritiated NAS (American Radiolabeled Chemicals, St. Louis, MO, USA) were added to monitor the HPLC retention profile, while deuterated internal standards consisting of 2 pmol of each deuterium-labeled neuroactive steroid (CDN Isotopes, Pointe-Claire, QC, and Steraloids, Newport, RI, USA) were used to allow quantification of the compound of interest and correct for procedural losses. Each steroid of interest was then derivatized in heptafluorobutyric acid (HFBA) (ThermoFisher, USA) for GC/MS (15, 35). Mass spectrometry analysis was performed in the standard electron impact mode for both ALLO and its isomers, and P4 measurements. The quantity of each NAS of interest was calculated by dividing the area under the peak of the NAS in the sample by the area under the peak of the deuterated internal standard. P4 sensitivity is ~.03 ng/mL with intra-assay coefficient of variation (CV) of ~4%. The detection limit for each NAS is approximately 10 fmol/mL. Intra-assay CV for these NAS is <5%.

### Analytic methods

NAS assays included in this analysis were run in three separate batches. The first batch utilized a different set of internal standards than batches two and three. We controlled for variance associated with these different batches and internal standards by extracting residuals from models predicting NAS values from these two factors, and using the extracted residuals as the primary NAS outcome variables. We then winsorized each residualized NAS variable by identifying any value above the 95^th^ percentile and setting those values to the value at the 95^th^ percentile. Primary figures therefore present residualized, winsorized NAS levels. For each NAS, an analysis of covariance (ANCOVA) for each NAS predicted a dependent variable of T1 NAS levels from the primary, 4-level categorical independent variable based on prior history of MDD (self-reported) and current T1 PND with levels coded as: 1) history of MDD and current T1 PND (*recurrent depression*), 2) history of MDD and no current T1 PND (*pre-pregnancy depression*), 3) no history of MDD and current T1 PND (*perinatal-emergent depression*), 4) no history of MDD and no current T1 PND (*never depressed*), controlling for EGA (in weeks) at the T1 visit. Four additional ANCOVAs predicted a dependent variable of T2 NAS levels from the categorized group variable with levels coded as: 1) history of MDD and either T1 or T2 PND (*recurrent depression*), 2) history of MDD and no T1 or T2 PND (*pre-pregnancy depression*), 3) no history of MDD but either T1 or T2 PND (*perinatal-emergent depression*), 4) no history of MDD and no T1 or T2 PND (*never depressed*), controlling for EGA (in weeks) at the T2 visit. Pairwise comparisons evaluated significant differences between groups. The threshold for significance in all statistical models was *p*=.05. Using G*Power, we determined that we had 80% power to detect conventionally small-to-medium effect sizes with our sample size and planned statistical models. Due to sample size constraints and statistical power concerns, our primary models did not include covariates except EGA. In sensitivity analyses, we included depression severity as measured by the CAT-DI module of the CAT-MH™ (36), as well as age, race, income, education, and Medicaid status as covariates in models. As this was a preliminary study in a small subset of participants from our parent study, we made no corrections for multiple comparisons so as to capture associations worthy of future analysis in a larger sample, and to generate future hypotheses.

## Results

### Participants

Participant characteristics are summarized in **Table 1**. Age differed significantly across history of depression/current depression groups (those who were never depressed were older than those with perinatal-emergent depression or recurrent depression), and was therefore included as a predictor variable in models for sensitivity analyses.

**Table 1:** Participant characteristics.

Variable	Perinatal- Emergent (N=18)	Recurrent Depression (N=10)	Pre-Pregnancy Depression (N=10)	Never Depressed (N=60)	Total (N=98)
Age in years, mean (SD)*	27.3 (7.2)	27.8 (5.5)	29.5 (4.7)	30.5 (5.4)	29.6 (5.9)
Range	18-39	20-41	22-36	19-41	18-41
Hispanic/Latina, *n* (%)	5 (27.8%)	5 (50.0%)	6 (60.0%)	16 (26.7%)	32 (32.7%)
Race, *n* (%)					
Caucasian/White	5 (27.8%)	4 (40.0%)	5 (50.0%)	20 (33.3%)	34 (34.7%)
Black	11 (61.1%)	5 (50.0%)	4 (40.0%)	36 (60.0%)	56 (57.1%)
American Indian	1 (5.6%)	1 (10.0%)	0 (0.0%)	1 (1.7%)	3 (3.1%)
Asian/Pacific Islander	0 (0.0%)	0 (0.0%)	1 (10.0%)	1 (1.7%)	2 (2.0%)
Unspecified	1 (5.6%)	0 (0.0%)	0 (0.0%)	2 (3.3%)	3 (3.1%)
Marital Status, *n* (%)					
Single	9 (50.0%)	6 (60.0%)	2 (20.0%)	20 (33.3%)	37 (37.8%)
Married/In Relationship	9 (50.0%)	4 (40.0%)	8 (80.0%)	40 (66.7%)	61 (62.2%)
Education, *n* (%)					
High school or GED	3 (16.7%)	1 (10.0%)	3 (30.0%)	20 (33.3%)	27 (27.6%)
Some college	9 (50.0%)	4 (40.0%)	2 (20.0%)	14 (23.3%)	29 (29.6%)
2 year college degree	0 (0.0%)	2 (20.0%)	1 (10.0%)	6 (10.0%)	9 (9.2%)
4 year college degree	4 (22.2%)	1 (10.0%)	1 (10.0%)	10 (16.7%)	16 (16.3%)
Master’s degree	2 (11.1%)	2 (20.0%)	2 (20.0%)	9 (15.0%)	15 (15.3%)
Professional degree/PhD	0 (0.0%)	0 (0.0%)	1 (10.0%)	1 (1.7%)	2 (2.0%)
Annual Income, *n* (%)					
<$15,000	8 (44.4%)	4 (40.0%)	4 (40.0%)	16 (26.7%)	32 (32.7%)
$15,000-$31,000	2 (11.1%)	3 (30.0%)	1 (10.0%)	17 (28.3%)	23 (23.4%)
$31,000 - $76,000	6 (33.3%)	3 (30.0%)	2 (20.0%)	15 (25.0%)	26 (26.5%)
$76,000 - $150,000	2 (11.1%)	0 (0.0%)	2 (20.0%)	10 (16.7%)	14 (14.3%)
>$150,000	0 (0.0%)	0 (0.0%)	1 (10.0%)	2 (3.3%)	3 (3.1%)
Federal Aid	14 (77.8%)	9 (90.0%)	5 (50.0%)	34 (56.7%)	62 (63.3%)
Multiparous	9 (50.0%)	6 (60.0%)	5 (50.0%)	40 (66.7%)	60 (61.2%)

* indicates group differences in demographic variable.

A total of 98 participants (57% Black, 33% Latina, 63% on Medicaid) contributed blood samples at the T1 visit. All 98 participants had detectable levels of P4; 94 had detectable levels of ALLO, PA, and ISO; 91 participants had detectable levels of EPI. A total of 93 participants contributed blood samples at the T2 visit. All 93 participants had detectable levels of P4; 91 had detectable levels of ALLO, PA, and EPI; 90 participants had detectable levels of ISO. Abnormal or non-detectable peaks were treated as missing data.

### First trimester

In the first trimester, 8 participants were recurrently depressed, 12 were depressed pre-pregnancy only, 13 had perinatal-emergent perinatal depression, and 65 were never depressed. Of the 20 total participants reporting a prior history of MDD, 6 reported a major depressive episode during a prior pregnancy or within the first year after a previous childbirth. In sensitivity analyses controlling for EGA, there were no differences in T1 levels of any NAS between those who reported a history of depression during or after a previous pregnancy versus those who were previously depressed outside of the perinatal period (see **Supplementary Table S1**).

**Figure 1:**
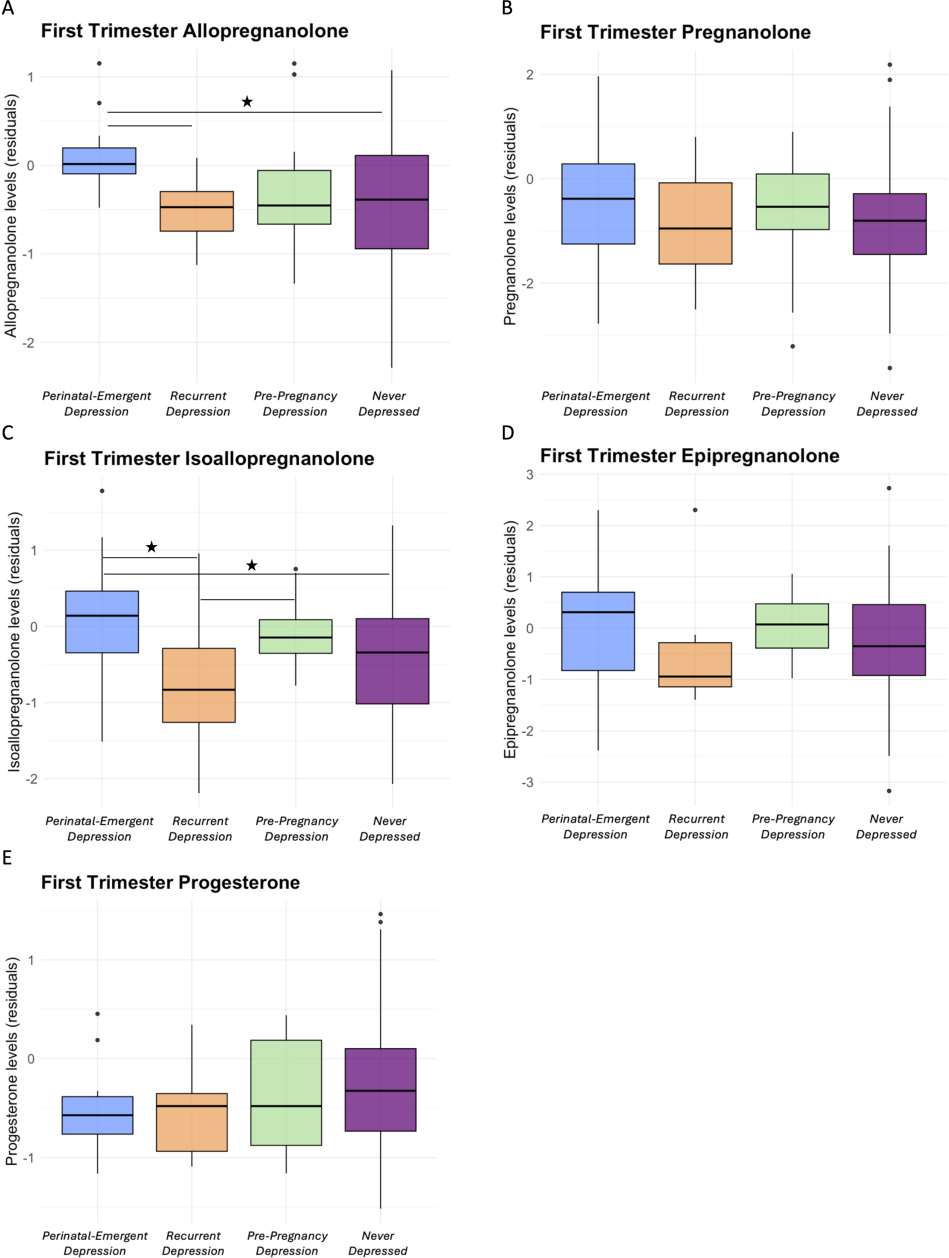
Residualized levels of allopregnanolone **(A)**, pregnanolone **(B)**, isoallopregnanolone **(C)**, epipregnanolone **(D)**, and progesterone **(E)** by history of depression and T1 depression status groups. *p<.05.

**Table 2** shows group differences (i.e., pairwise comparisons) in NAS levels in T1. Across the four categorized groups, there were marginally significant differences in T1 ALLO levels (*p*=.05) and T1 ISO levels (*p*=.05). Pairwise comparisons showed that ALLO levels were significantly higher in those with perinatal-emergent depression compared to those who were never depressed (**Figure 1A**; *p*=.007), and marginally significantly higher than those with recurrent depression (*p*=.05). ISO levels were higher in those with perinatal-emergent depression compared to both those who were never depressed (*p*=.03) and those with recurrent depression (*p*=.02; **Figure 1C**). Effect sizes for significant effects were small-to-medium (Cohen’s F^2^>0.08). There were no other significant pairwise differences in NAS levels between groups (all *p*s>.10). There were no other significant pairwise differences in NAS levels between groups (all ps>.10; **Figures 1B, D, E**). In sensitivity analyses including depression severity and sociodemographic variables as covariates, results remained largely unchanged (**Supplementary Table S2**).

**Table 2:** Results from ANCOVA and pairwise comparisons of T1 NAS levels across four groups based on a history of MDD and current PND.

Reference Group/Comparison	*Dependent Variable:*				
	ALLO	PA	ISO	EPI	P4
Perinatal Emergent/Never Depressed	-0.56* (0.20)	-0.50 (0.35)	-0.52* (0.24)	-0.23 (0.34)	0.30 (0.21)
Perinatal Emergent/Recurrent Depression	-0.58’ (0.29)	-0.52 (0.52)	-0.81* (0.35)	-0.32 (0.54)	0.06 (0.30)
Perinatal Emergent/Pre-Pregnancy	-0.34 (0.26)	-0.36 (0.46)	-0.17 (0.31)	0.09 (0.44)	0.19 (0.27)
Never Depressed/Recurrent Depression	-0.03 (0.25)	-0.02 (0.43)	-0.29 (0.29)	-0.09 (0.46)	-0.24 (0.25)
Never Depressed/Pre-Pregnancy	0.22 (0.21)	0.14 (0.37)	0.35 (0.25)	0.32 (0.34)	-0.11 (0.21)
Recurrent Depression/Pre-Pregnancy	0.24 (0.30)	0.16 (0.52)	0.64’ (0.36)	0.41 (0.54)	0.13 (0.30)
Observations	94	94	94	91	98
Group - *F Value*	2.71’	0.68	2.61’	0.46	1.05
EGA - *F Value*	2.83’	1.08	0.14	0.06	5.44*

’0.5<p<0.1; *p<.05; unless otherwise noted, presented values are beta values and standard errors as *b*(SE).

### Second trimester

In the second trimester, 10 participants had recurrent depression (at either T1 or T2), 8 had pre-pregnancy depression only, 17 had perinatal-emergent depression (at either T1 or T2), and 58 were never depressed. Of the 18 total participants reporting a prior history of MDD, 6 reported a previous diagnosis of depression during pregnancy or the first year after childbirth. In sensitivity analyses controlling for EGA, there were no differences in T2 levels of any NAS between those who reported a prior history of depression during or after a previous pregnancy versus those who were previously depressed outside of the perinatal period (see **Supplementary Table S3**).

**Figure 2:**
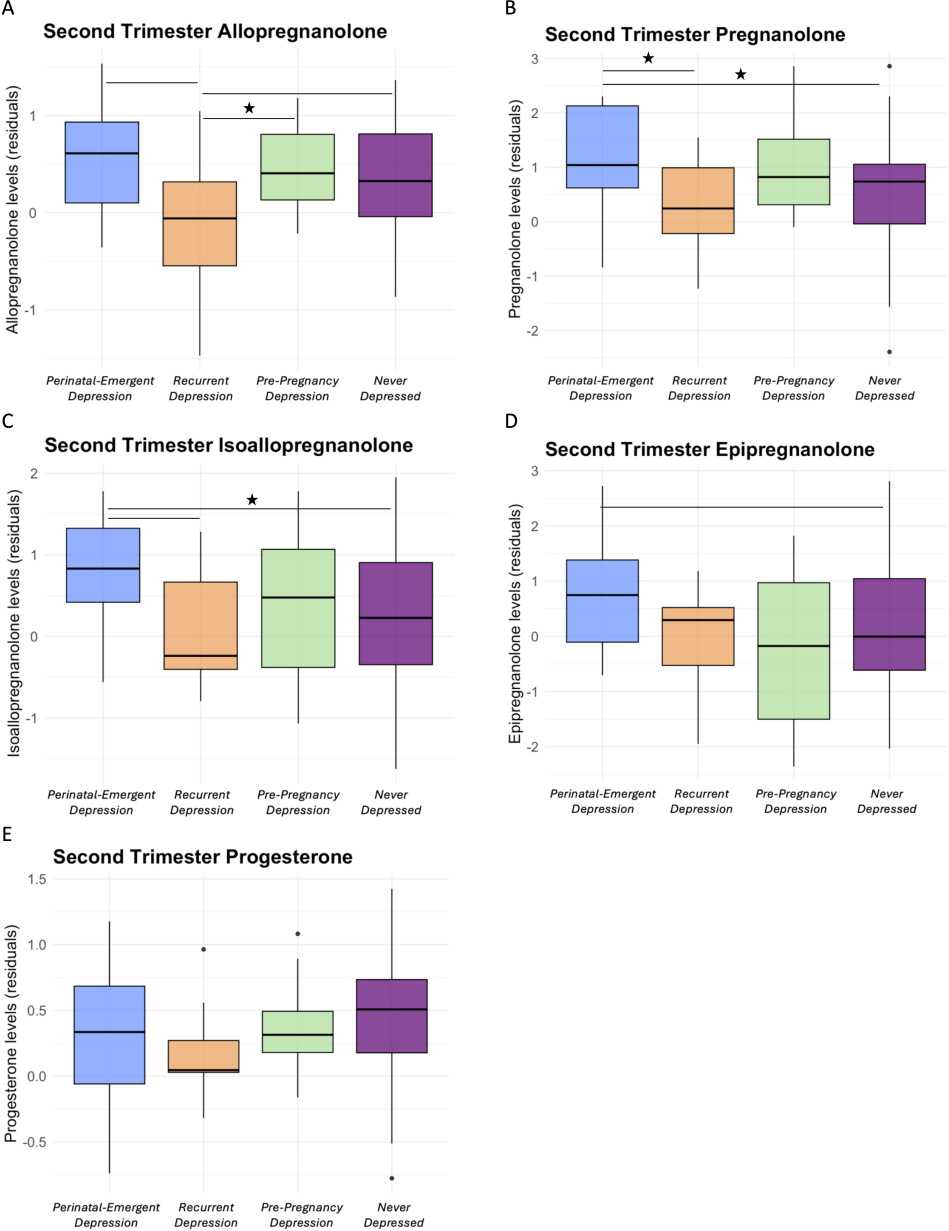
Residualized levels of allopregnanolone **(A)**, pregnanolone **(B)**, isoallopregnanolone **(C)**, epipregnanolone **(D)**, and progesterone **(E)** by history of depression and T2 depression status groups. *p<.05.

Across the four groups, there were significant differences in T2 PA levels (*p*=.04), and marginally significant differences in T2 ALLO levels (*p*=.07) and ISO levels (*p*=.09). **Table 3** shows group differences (i.e., pairwise comparisons) in NAS levels in T2. Pairwise comparisons showed that ALLO levels were marginally significantly higher in those with perinatal-emergent depression compared to those with recurrent depression (**Figure 2A**; *p*=.05). ALLO levels were also marginally significantly higher in those who were never depressed (*p*=.05) or had depression only pre-pregnancy (*p*=.04) compared to those with recurrent depression. PA levels were significantly higher in those with perinatal-emergent depression compared to both those who were never depressed (*p*=.02) and those with recurrent depression (**Figure 2B**; *p*=.01). Further, levels of ISO were significantly higher in those with perinatal-emergent depression compared to those who were never depressed (*p*=.03) and marginally higher than those with recurrent depression (**Figure 2C**; p=.07). EPI (*p*=.09) levels were also marginally significantly higher in those with perinatal-emergent depression compared to those who were never depressed. Effect sizes for significant effects were small-to-medium (Cohen’s F^2^>0.08). There were no other significant differences in NAS between groups (all ps>.10; **Figures 2D, E**). There were also expected increases in ALLO (*p*=.05) and P4 (*p*=.04) across EGA weeks in the second trimester. In sensitivity analyses including depression severity and sociodemographic variables as covariates, results remained largely unchanged (**Supplementary Table S4**).

**Table 3:** Results from ANCOVA and pairwise comparisons of T2 NAS levels across four groups based on a history of MDD and current PND.

Reference Group/Comparison	*Dependent Variable:*				
	ALLO	PA	ISO	EPI	P4
Perinatal Emergent/Never Depressed	-0.07 (0.17)	-0.67* (0.27)	-0.50* (0.23)	-0.56’ (0.33)	0.24’ (0.13)
Perinatal Emergent/Recurrent Depression	-0.48’ (0.24)	-1.00* (0.40)	-0.63’ (0.35)	-0.75 (0.48)	0.04 (0.19)
Perinatal Emergent/Pre-Pregnancy	-0.08 (0.26)	-0.26 (0.43)	-0.39 (0.37)	-0.83 (0.52)	0.25 (0.20)
Never Depressed/Recurrent Depression	-0.40’ (0.20)	-0.33 (0.33)	-0.13 (0.29)	-0.19 (0.40)	-0.20 (0.16)
Never Depressed/Pre-Pregnancy	0.16 (0.22)	0.41 (0.37)	0.11 (0.31)	-0.27 (0.45)	0.01 (0.17)
Recurrent Depression/Pre-Pregnancy	0.56* (0.28)	0.74 (0.46)	0.24 (0.40)	-0.08 (0.55)	0.21 (0.22)
Observations	91	91	90	91	93
Group - *F Value*	2.46’	2.87*	2.28’	2.17	1.17
EGA - *F Value*	3.89’	0.30	0.71	1.64	3.99*

’0.5<p<0.1; *p<.05; unless otherwise noted, presented values are beta values and standard errors as*b*(SE).

## Discussion

This study is the first, to our knowledge, to directly examine whether levels of ALLO or other P4-derived NAS in pregnancy differ based on the timing of depression onset before and/or during early pregnancy. We found that, in general, NAS levels were higher in those with *perinatal-emergent* depression (i.e., first depressive episode in the first or second trimester) compared to those who had recurrent depression (pre-pregnancy and during early pregnancy) and those who were never depressed. Specifically, those with perinatal-emergent depression had higher ALLO and ISO levels in the first trimester compared to those with recurrent depression and those who were never depressed. Those with perinatal-emergent depression had higher ALLO, PA, and ISO levels in the second trimester compared to those with recurrent depression, and had higher T2 PA and ISO levels compared to those who were never depressed. T2 ALLO levels in those with recurrent depression were also lower than ALLO levels in those with pre-pregnancy depression only or those who were never depressed. These findings are somewhat consistent with the limited prior literature, where those with a prior history of MDD displayed lower concentrations of ALLO and PA both before and after an oral progesterone challenge compared to those who had never been depressed (30). In the present study, those with *perinatal-emergent* depression had higher concentrations of ALLO, ISO and PA compared to those who had *recurrent* depression, but not compared to those who had *pre-pregnancy depression* only. Therefore, a history of depression alone may not predict NAS levels during pregnancy – whether it persists into pregnancy appears to be an important, interacting factor.

This analysis of NAS by both pre-pregnancy history of MDD *and* MDD during pregnancy suggests that the interaction of both factors may be important in investigating the pathogenesis of PND and in understanding some of the variability across previous studies in the presence and direction of association between NAS and antenatal depression (4–8, 10, 37). These findings add to the limited literature on NAS in early pregnancy, though future research should expand investigation of these group differences into the third trimester and postpartum period, as these relationships may differ between periods of hormone surge (early pregnancy) and hormone withdrawal (following delivery). Further, this is the first study to examine this association with ISO, a negative modulator at the GABA_A_R, and EPI, which antagonizes GABA_A_R at high concentrations but was also found to potentiate GABA-induced chloride current at a low concentration range in native neurons obtained from rat cerebellar or hippocampal slices (38). While ISO was significantly higher at T1 in those with perinatal-emergent depression, there were no significant differences in EPI between groups. ISO is an antagonist to ALLO and PA action at the GABA_A_R (17), but its relationship to PND is not well understood. Generally, our findings suggest that perinatal-emergent depression may be associated with upregulated synthesis of the positive GABA_A_R modulators ALLO and PA, and to a lesser extent the negative GABA_A_R modulators. This pattern could indicate preferential action of the 3β-HSD enzyme on metabolism of 5α-DHP to ISO, rather than 5β-DHP to EPI, particularly in those with perinatal-emergent depression. Future research including measurements of NAS, their precursors (5α-DHP and 5β-DHP), and studies on the expression of enzymes involved in the P4 metabolic pathway will be important in elucidating specific mechanisms of action.

These findings suggest pathophysiological heterogeneity in PND, and may be interpreted in a number of ways. First, differences between those with recurrent depression and those with perinatal-emergent depression may represent differences in neurosteroidogenesis based on chronic or acute stress states. Chronic and acute stressors differentially impact neurosteroidogenesis. While chronic stress and chronic MDD are associated with blunted neurosteroidogenesis, acute stressors are associated with upregulated neurosteroidogenesis (22–28). Our findings may suggest that upregulated neurosteroidogenesis resulting in higher T1 and/or T2 levels of NAS (a greater early pregnancy surge) in those with perinatal-emergent depression may be a response to new onset depression with no prior history of MDD, a characteristically “acute” stress state. Chronic or recurrent MDD, as is the case of those who have a prior history of depression and remain depressed during pregnancy, may result in persistently blunted neurosteroidogenesis, and low levels of NAS, that continues into pregnancy. Additionally, postpartum depression has been associated with alterations in the expression of GABA_A_ receptor subunits, which play a major role in determining receptor sensitivity to NAS (39). Importantly, subunit expression is extremely plastic, changing in response to fluctuating NAS (40–42). For example, rodent models suggest that expression of the δ subunit, which is the preferred target of NAS, is downregulated during pregnancy (a hypothesized compensatory mechanism to offset high, potentially sedative, levels of ALLO) and rebounds postpartum (39). Further, in a δ receptor knockout model, female mice display depression-like behaviors and abnormal maternal behaviors postpartum (39). A combination of fluctuating NAS levels (i.e., surging *or* blunted neurosteroidogenesis) and suboptimal adaptation of GABA_A_ receptor subunit expression during pregnancy could contribute to disrupted NAS-mediated inhibitory signaling, contributing to elevated mood symptoms. Future work examining GABA_A_ receptor subunit expression in relation to NAS levels in the groups examined in the present study would inform our understanding of these mechanisms.

Alternatively, those with perinatal-emergent depression during early pregnancy may represent a clinical phenotype of differential NAS change in which *a greater early pregnancy surge in NAS triggers depressive symptoms*, rather than the onset of depression triggering an increase in NAS. These hypotheses could also work in tandem, whereby those with a history of chronic MDD enter pregnancy with blunted neurosteroidogenesis and experience a somewhat blunted surge in early pregnancy, while a subset of others may have no prior history of MDD but may be sensitive to a large early pregnancy surge in sex steroid hormones and NAS, inducing depressive symptoms. In this case, two separate clinical phenotypes of PND may be present, each with its own unique NAS-related pathophysiology. While one group may represent a negative mood response to a greater early pregnancy surge in NAS (similar to luteal-phase sensitivity to surging P4/ALLO seen in some individuals with premenstrual dysphoric disorder), the other may represent a chronic, negative mood response to persistently low NAS.

While this study contributes novel findings to the literature, it has limitations. First, we only assessed NAS levels during the first and second trimesters. In ongoing studies we are assessing NAS across the entire perinatal period using the group categorizations explored here, but the addition of third trimester and postpartum blood draws in our study protocol occurred more recently, and we do not yet have a sufficient sample size to assess this relationship across the entire pregnancy and postpartum period. However, the present analysis will be critical in providing a comprehensive understanding of how a prior history of MDD and timing of PND onset influence NAS across the entire perinatal period, as NAS levels continue to fluctuate later in pregnancy and decline in the postpartum. Second, the self-reported nature of our MDD history classification is inherently limited. A structured clinical diagnostic interview is the gold-standard for determining a comprehensive prior history of mental health disorders, and future studies should aim to include these to increase confidence in grouping participants by mental health history. Relatedly, we screened for MDD during pregnancy using the CAT-MH, and while this is validated in perinatal populations (32) and in our own cohort (31), a structured clinical diagnostic interview would be preferred in determining presence of a major depressive episode. The sample size for some groups was also quite small (i.e., N=8 with recurrent depression at T1). Analysis of a larger sample would increase confidence in the reliability our findings, though we emphasize the novelty of these examined associations and their relevance in generating hypotheses for future work. We also limited our inclusion of covariates due to sample size constraints, but recognize the importance of considering how other factors such as sociodemographic variables, comorbid psychiatric diagnoses, and trauma may influence these relationships. Additionally, though we accounted for it statistically, the use of data from different assay batches, and particularly the use of two different internal standards, is a limitation. Finally, the second trimester blood draw sometimes co- occurred with a 1-hour glucose challenge test for gestational diabetes, and while there are no data to suggest an effect of this test on levels of NAS or P4, this is a potential confound.

In conclusion, this work provides novel data on how a pre-pregnancy history of depression *and* time of onset of depression during early pregnancy may influence the relationship between NAS and PND. Future research should account for these factors, recognizing potential differences in pathophysiology for different clinical phenotypes of PND, and informing novel methods of prevention and treatment across dimensional groups.

## Data Availability

The raw data supporting the conclusions of this article will be made available by the authors, without undue reservation.
