# Supplementary material for: Levels of neuroactive steroids are elevated in those who develop first-onset depression early in pregnancy
Source: Front Psychiatry. 2025 May 8;16:1557560. doi: 10.3389/fpsyt.2025.1557560 (PMC12095207; doi:10.3389/fpsyt.2025.1557560)
Supplement: Supplementary file 1 [file Table1.docx]

**Supplementary Table 1.** In those reporting a prior history of MDD, comparison of T1 NAS in those who reported that this was during or after a prior pregnancy or those who reported a non-perinatal history of MDD.

|  | *Dependent Variable:* | | | | |
| --- | --- | --- | --- | --- | --- |
|  | ALLO | PA | ISO | EPI | P4 |
| Observations | 20 | 20 | 20 | 20 | 20 |
| Hx PND - *F Value* | 2.97 | 0.46 | 1.29 | 1.03 | 0.37 |
| EGA - *F Value* | 3.63’ | 5.54* | 0.81 | 1.38 | 2.02 |

*Note:* ‘0.5<p<0.1; *p<.05; **p<.01

**Supplementary Table 2.** Sensitivity analyses of T1 group differences in NAS, controlling for depression severity, age, race, income, education, and Medicaid status.

|  | *Dependent Variable:* | | | | |
| --- | --- | --- | --- | --- | --- |
| **Reference Group/Comparison** | ALLO | PA | ISO | EPI | P4 |
| Perinatal Emergent/Never Depressed | -0.45 (0.31) | -0.20 (0.56) | 0.10 (0.37) | -0.20 (0.57) | 0.01 (0.31) |
| Perinatal Emergent/Recurrent Depression | -0.65* (0.30) | -0.58 (0.55) | -1.01** (0.36) | -0.23 (0.61) | 0.06 (0.31) |
| Perinatal Emergent/Pre-Pregnancy | -0.18 (0.31) | 0.02 (0.56) | 0.27 (0.37) | 0.15 (0.57) | -0.03 (0.31) |
| Never Depressed/Recurrent Depression | -0.21 (0.38) | -0.37 (0.69) | -1.11* (0.46) | -0.04 (0.73) | 0.05 (0.38) |
| Never Depressed/Pre-Pregnancy | 0.27 (0.23) | 0.22 (0.42) | 0.16 (0.28) | 0.35 (0.42) | -0.04 (0.24) |
| Recurrent Depression/Pre-Pregnancy | 0.47 (0.37) | 0.60 (0.67) | 1.28** (0.44) | 0.39 (0.71) | -0.09 (0.37) |
| Observations | 94 | 94 | 94 | 91 | 98 |
| MDD Hx/T1 MDD - *F Value* | 2.59’ | 0.72 | 2.74* | 0.47 | 1.29 |
| EGA - *F Value* | 2.09 | 0.74 | 0.21 | 0.01 | 7.18** |
| CAT-DI Severity – *F Value* | 0.003 | 0.66 | 3.84’ | 0.02 | 0.10 |
| Age – *F Value* | 1.39 | 0.23 | 2.38 | 0.67 | 5.50* |
| Income – *F Value* | 3.70’ | 1.98 | 1.35 | 0.07 | 0.37 |
| Education – *F Value* | 5.77* | 0.16 | 2.61 | 0.13 | 0.25 |
| Race – *F Value* | 0.47 | 1.07 | 0.68 | 0.17 | 0.52 |
| Medicaid Status – *F Value* | 0.42 | 0.29 | 1.76 | 0.66 | 1.41 |

*Note:* ‘0.5<p<0.1; *p<.05; **p<.01; unless otherwise noted, presented values are beta values and standard errors as *b*(SE).

**Supplemental Table 3.** In those reporting a prior history of MDD, comparison of T2 NAS in those who reported that this was during or after a prior pregnancy or those who reported a non-perinatal history of MDD.

|  | *Dependent Variable:* | | | | |
| --- | --- | --- | --- | --- | --- |
| **Hx of Perinatal Depression** | ALLO | PA | ISO | EPI | P4 |
| Observations | 18 | 18 | 17 | 18 | 17 |
| Hx PND - *F Value* | 0.00 | 0.89 | 0.00 | 0.93 | 0.60 |
| EGA - *F Value* | 3.85’ | 0.67 | 0.24 | 0.72 | 8.92** |

*Note:* ‘0.5<p<0.1; *p<.05; **p<.01

|  | *Dependent Variable:* | | | | |
| --- | --- | --- | --- | --- | --- |
| **Reference Group/Comparison** | ALLO | PA | ISO | EPI | P4 |
| Perinatal Emergent/Never Depressed | 0.02 (0.21) | -0.53 (0.37) | -0.35 (0.31) | -0.33 (0.44) | 0.15 (0.17) |
| Perinatal Emergent/Recurrent Depression | -0.65* (0.26) | -1.13* (0.44) | -0.65’ (0.38) | -0.63 (0.53) | 0.01 (0.20) |
| Perinatal Emergent/Pre-Pregnancy | 0.17 (0.30) | -0.03 (0.52) | -0.09 (0.43) | -0.35 (0.62) | 0.03 (0.24) |
| Never Depressed/Recurrent Depression | -0.67* (0.27) | -0.61 (0.47) | -0.30 (0.40) | -0.31 (0.57) | -0.15 (0.22) |
| Never Depressed/Pre-Pregnancy | 0.15 (0.25) | 0.49 (0.42) | -0.26 (0.36) | -0.03 (0.51) | -0.13 (0.20) |
| Recurrent Depression/Pre-Pregnancy | 0.82* (0.33) | 1.10 (0.57) | 0.56 (0.49) | 0.28 (0.69) | 0.19 (0.27) |
| Observations | 91 | 91 | 90 | 91 | 93 |
| MDD Hx/T2 MDD - *F Value* | 2.37’ | 2.64’ | 2.12’ | 1.62 | 0.94 |
| EGA - *F Value* | 3.92’ | 0.11 | 1.47 | 2.70 | 2.49 |
| CAT-DI Severity – *F Value* | 3.78’ | 0.62 | 0.36 | 0.74 | 0.54 |
| Age – *F Value* | 1.34 | 0.04 | 0.00 | 0.02 | 0.52 |
| Income – *F Value* | 0.31 | 0.03 | 0.06 | 0.03 | 0.62 |
| Education – *F Value* | 0.89 | 0.02 | 0.49 | 0.10 | 2.89’ |
| Race – *F Value* | 1.82 | 0.04 | 0.23 | 0.71 | 1.36 |
| Medicaid – *F Value* | 1.85 | 0.98 | 0.08 | 1.17 | 0.60 |

**Supplemental Table 4.** Sensitivity analyses of T2 group differences in NAS, controlling for depression severity, age, race, income, education, and Medicaid status.

*Note:* ‘0.5<p<0.1; *p<.05; **p<.01; unless otherwise noted, presented values are beta values and standard errors as *b*(SE).
